# Topical Application of Fasudil Hydrochloride for Vasospasm during Soft Tissue Reconstruction Using a Free Flap

**DOI:** 10.1155/2019/5929281

**Published:** 2019-04-28

**Authors:** Kaoru Tada, Tadahiro Nakajima, Mika Nakada, Masashi Matsuta, Hiroyuki Tsuchiya

**Affiliations:** Department of Orthopaedic Surgery, Graduate School of Medical Science, Kanazawa University, 13-1 Takaramachi, Kanazawa 920-1302, Japan

## Abstract

Vasospasm is a phenomenon that can complicate microsurgery. We report a case in which vasospasm was quickly relieved by topical application of fasudil hydrochloride. A 36-year-old man underwent preoperative chemotherapy and wide excision for a malignant soft tissue tumor around the knee joint. We planned reconstruction using a free latissimus dorsi muscle flap for the resulting soft tissue defect and selected the peroneal vessels as the recipient vessels. However, there was no arterial blood flow from the peroneal vessels, which we diagnosed as vasospasm. Conventional treatment of the vasospasm was attempted, but blood flow was not achieved. Topical application of fasudil hydrochloride solution promptly relieved the vasospasm. To the best of our knowledge, this is the first clinical report of the use of fasudil hydrochloride for vasospasm during soft tissue reconstruction using a free flap.

## 1. Introduction

A phenomena that may possibly occur while performing soft tissue reconstruction using a free flap is vasospasm of the recipient vessels. It is thought that vasospasm is caused by abnormal contraction of vascular smooth muscle. Such vasospasm is mainly caused by intraoperative dissection and manipulation of the vessels and is estimated to occur in 5%–10% of all microsurgical procedures [1]. As treatment for vasospasm, topical administration of lidocaine and papaverine hydrochloride and methods involving warming up the vessels have been reported but are not necessarily effective. In cases in which the vasospasm is not relieved, it is necessary to undertake measures, such as reanastomosis of the vessels, more proximal vascular anastomosis, or preparation of other recipient vessels. Here, we report a case in which the vasospasm was quickly relieved by topical application of fasudil hydrochloride. This is possibly the first clinical report of the use of fasudil hydrochloride for vasospasm during soft tissue reconstruction using a free flap.

## 2. Case Report

A 36-year-old man was diagnosed with synovial sarcoma around the left knee joint. After 4 courses of preoperative chemotherapy, we planned wide excision including the peroneal nerve and fibular head. We planned reconstruction of the resulting soft tissue defect using a free latissimus dorsi muscle flap and reconstruction of the peroneal nerve defect using a sural nerve graft. For the recipient vessels, the peroneal artery and vein were selected after confirming patency by enhanced computed tomography and ultrasonography.

**Figure 1:**
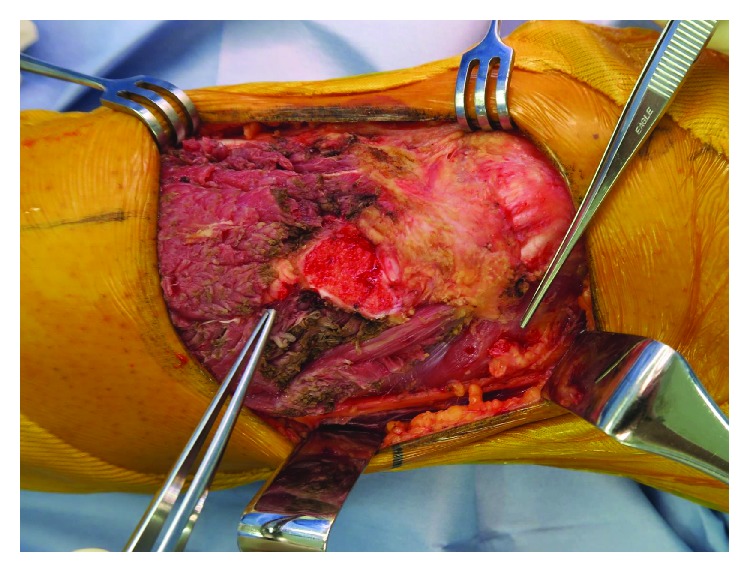
The size of the soft tissue defect after wide excision was 11 × 13 cm.

**Figure 2:**
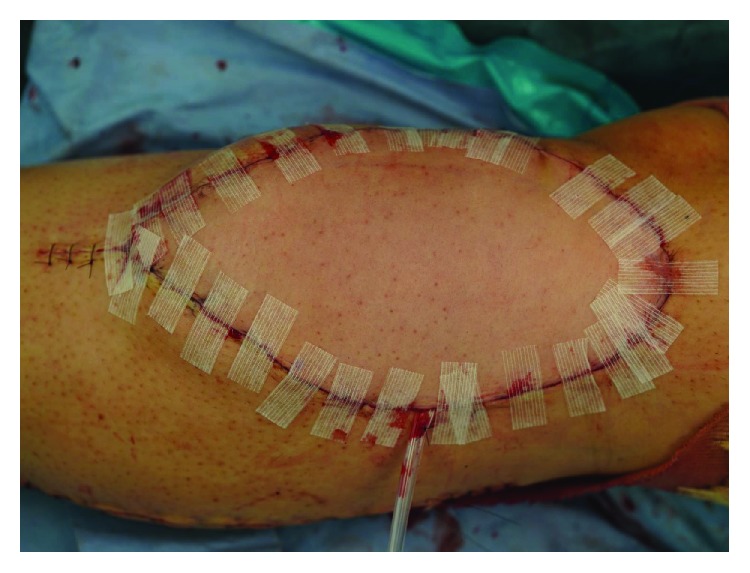
The wound was sutured after confirming there was no bleeding point in the operative field.

The size of the soft tissue defect after wide excision was 11 × 13 cm (Figure 1). Thus, an 11 × 15 cm latissimus dorsi muscle flap was elevated. When the recipient vessels were incised after dissection, arterial blood flow was not observed. We diagnosed vasospasm and attempted to warm up the vessels using warm saline, along with topical application of heparin solution and 2% lidocaine. However, no arterial blood flow was achieved after 15 minutes. Subsequently, we sprayed approximately 5 mL of a 15-fold dilution of fasudil hydrochloride (ERIL^TM^, Asahi Kasei Pharma, Japan) with saline around the recipient vessels. Arterial blood flow and pulsation appeared soon after application, and arterial blood spouting was achieved after approximately 1 minute. Thereafter, 1 artery and 2 veins were anastomosed, and the wound was sutured after confirming there was no bleeding point in the operative field (Figure 2). No complications, such as wound hemorrhage or hematoma formation, were observed after surgery, and the flap completely survived.

## 3. Discussion

Vasospasm remains a possible complication during soft tissue reconstruction using a free flap, and the frequency is reported to be particularly high in reconstruction of the extremities [2]. Warming up the vessels and topical application of lidocaine, papaverine hydrochloride, or calcium channel blocker have been reported as the possible treatments for vasospasm [3]. A recent report revealed that >90% of microsurgeons use vasodilators during microsurgery according to their preference [4].

However, even with these vasodilators, cases in which vasospasm is not relieved are often experienced, and in our case, no obvious effect was found. In a recent systematic review, Vargas et al. reported that the available literature on the use of topical vasodilators for the intraoperative management of vasospasm during microsurgery is limited and largely based on animal models, which may not be reliably generalizable to the reconstructive patient population [5].

Fasudil hydrochloride can effectively relax vascular smooth muscle by inhibiting Rho kinase selectively [6] and has been used for the prevention of cerebral vasospasm after surgery for subarachnoid hemorrhage [7]. Furthermore, there have been some reports about its effectiveness against vasospasm of the coronary artery [8]. Although fasudil hydrochloride has a strong vasodilatory effect, its half-life is only approximately 15 minutes. Therefore, the possibility of postoperative bleeding and hematoma formation is likely to be low as a result of topical application, and no complications were observed in our case.

Our case did not have a history of trauma around the lower limb. Therefore, the vasospasm was thought to be transient and caused by mechanical stimulation rather than persistent and caused by posttraumatic vessel disease. The vasospasm might have been relieved by continuing conventional treatment (warming up the vessel and application of lidocaine), but the time to wait for such relief can be unacceptably long for the microsurgeon. Since the onset of the effect of fasudil hydrochloride was extremely rapid, it is considered to be a viable treatment option for vasospasm during soft tissue reconstruction using a free flap. Although future studies are required to determine the proper dosage and concentration, the results of our case suggest that fasudil hydrochloride is effective against vasospasm.
